# Chitosan-gated organic transistors printed on ethyl cellulose as a versatile platform for edible electronics and bioelectronics†

**DOI:** 10.1039/d3nr01051a

**Published:** 2023-05-22

**Authors:** Alina S. Sharova, Francesco Modena, Alessandro Luzio, Filippo Melloni, Pietro Cataldi, Fabrizio Viola, Leonardo Lamanna, Nicolas F. Zorn, Mauro Sassi, Carlotta Ronchi, Jana Zaumseil, Luca Beverina, Maria Rosa Antognazza, Mario Caironi

**Affiliations:** a Center for Nano Science and Technology, Istituto Italiano di Tecnologia Via Raffaele Rubattino 81 20134 Milano Italy mario.caironi@iit.it; b Department of Physics, Politecnico di Milano Piazza Leonardo da Vinci 32 20133 Milano Italy; c Department of Electronics, Information and Bioengineering, Politecnico di Milano Piazza Leonardo da Vinci 32 20133 Milano Italy; d Smart Materials, Istituto Italiano di Tecnologia Via Morego 30 16163 Genova Italy; e Institute for Physical Chemistry, Heidelberg University 69120 Heidelberg Germany; f Department of Materials Science, Università degli Studi di Milano-Bicocca via Cozzi 55 20125 Milano Italy; g Department of Engineering for Innovation, University of Salento Via per Monteroni 73100 Lecce Italy

## Abstract

Edible electronics is an emerging research field targeting electronic devices that can be safely ingested and directly digested or metabolized by the human body. As such, it paves the way to a whole new family of applications, ranging from ingestible medical devices and biosensors to smart labelling for food quality monitoring and anti-counterfeiting. Being a newborn research field, many challenges need to be addressed to realize fully edible electronic components. In particular, an extended library of edible electronic materials is required, with suitable electronic properties depending on the target device and compatible with large-area printing processes, to allow scalable and cost-effective manufacturing. In this work, we propose a platform for future low-voltage edible transistors and circuits that comprises an edible chitosan gating medium and inkjet-printed inert gold electrodes, compatible with low thermal budget edible substrates, such as ethylcellulose. We report the compatibility of the platform, characterized by critical channel features as low as 10 μm, with different inkjet-printed carbon-based semiconductors, including biocompatible polymers present in the picogram range per device. A complementary organic inverter is also demonstrated with the same platform as a proof-of-principle logic gate. The presented results offer a promising approach to future low-voltage edible active circuitry, as well as a testbed for non-toxic printable semiconductors.

## Abstract 2

A platform for edible transistors is presented, comprising a chitosan-based gating medium, an ethylcellulose substrate, and printed gold electrodes.

## Graphical abstract

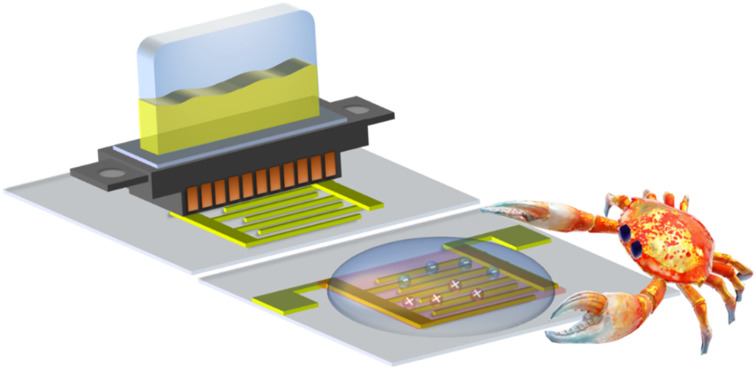

## 1. Introduction

Active attempts have been made over the last few years towards the realization of “benign” electronic devices, employing materials of sustainable natural origin and biodegradable,^1^ biocompatible,^2^ green,^3^ and organic (carbon-based)^4,5^ in the design. The emerging field of edible electronics,^6,7^ among others, goes past the traditional model of electronic devices and exploits the inherent electronic properties of food, food-derived, or edible synthetic materials for the development of functional electronic components and systems that can be safely ingested and subsequently digested after accomplishing their task.^8,9^ Despite offering cutting-edge opportunities for biomedicine, pharmaceuticals, and the food industry, this field is currently facing tremendous challenges, particularly from the point of view of materials selection and their energy efficient processing.^10–12^ The specific nature of soft edible materials, having comparatively poor chemical and thermal stability, poses limitations on the use of conventional deposition and fabrication techniques (*e.g.*, photolithography).

In this context, inkjet printing represents an appealing fabrication platform for large-area, scalable and cost-effective edible electronics. Inkjet printing can enable rapid prototyping and direct patterning of materials selectively on the target region, at low temperatures and on various flexible, conformable or edible substrates.^13^ Being an additive process, inkjet printing allows drastic reduction in the consumption of costly ink materials. Therefore, it decreases the overall cost of a single device and minimizes the amount of material per device upon ingestion. However, the critical dimensions obtained by inkjet printing are generally restricted to a few tens to a few hundreds of micrometers.^2,6,8^

Transistors are the building blocks of digital and analog electronic circuits and constitute the key elements to be developed within the framework of edible and sustainable electronics. These microelectronic devices are necessary to provide the fundamental electronic duties of tracking, monitoring, sensing, and data transmission, and serve, therefore, as a backbone platform for future electronic systems. Owing to their multi-layered structure, transistors are relatively complex functional elements to be developed. Thus, different classes of edible materials constituting the device (*i.e.*, conductors, dielectrics, semiconductors) need to be selected and assembled accordingly.

Within the framework of edible electronics, low-voltage (<1 V) operation is essential, and the use of electrolytes (*i.e.*, electronic insulating, but ionic conducting materials) as gate dielectrics is a powerful option. Pure/distilled water (self-ionized into hydronium and hydroxide ions) represents a common gating medium for organic transistors.^14^ In this configuration, the application of a gate bias causes the ions present in the electrolyte to drift at the interfaces formed with both the gate and the semiconductor. If these ionic species are not able to permeate the semiconductor, an electric double layer (EDL) is formed at the interface. This can be modelled as a nanometric capacitor, able to provide large area capacitance values (μF cm^−2^), which can be exploited for inducing high sheet carrier densities (10^14^ cm^−2^) within the semiconductor and operating the transistor at very low voltages (<1 V). In addition, electrolyte-gated transistors feature simple and versatile architectures and stability in aqueous environments which, together with the ability to provide coupling of both ionic and electronic domains in a single device, pave the way to several new opportunities for future sensing and biosensing applications.^15,16^

Among various electrolytes (*e.g.*, ionic liquids, ion gels, aqueous and solid-state electrolytes), solid-state electrolytes potentially provide essential advantages for practical application in terms of material stability, resistance to physical damage and the possibility of miniaturized fabrication down to the scale of thin films. Solid electrolytes are also appealing as they are solution processable and printable, but retain a sufficient mechanical robustness to allow for the design of more complex architectures, and possibly for integrated circuitry for flexible, stretchable, and conformable edible electronics.^17–19^

In this work, we exploit the electronic properties of edible substrates, electrodes, and gating media for fully printed electrolyte-gated transistors. First, we demonstrate the use of inkjet-printed inert gold electrodes on temperature-sensitive edible substrates. The inkjet-printing process optimized for a water-based gold ink, and a detailed morphological, structural, and electrical characterization of the final interdigitated patterns on different conventional and edible substrates are provided. Fully printed water-gated field-effect transistors (WGFETs) are further realized to assess the electronic performances and the quality of the electrodes, in combination with two model carbon-based semiconductors.

Next, we introduce an inherently safe and edible compound, chitosan, as an effective solid-state gating medium for the realization of air-stable organic transistors operating at low voltage (<1 V). P-type and n-type inkjet-printed chitosan-gated transistors along with complementary inverting logic gates are demonstrated on edible and flexible ethyl cellulose substrates and can be easily integrated into temporary ingestible form factors, such as pills or capsules. Overall, the proposed devices, here demonstrated with reference carbon-based semiconductors, combining edible substrates, inkjet-printed inert gold electrodes, and an edible electrolyte gating medium, offer a reference platform for the development of future fully edible transistors and circuits.

## 2. Results and discussion

### 2.1. Inkjet printed gold electrodes

Conducting electrodes are an essential part of electronic devices and their quality and spatial resolution are of crucial importance for the performances of organic transistors and circuits. Among the various conductive metals available, gold is the most common and extensively utilized material for their fabrication. Gold is an edible and biologically inert substance with high electrical conductivity, chemical stability, and proven biocompatibility.^20^ It has excellent resistance to oxidation and acids and it is the preferred option for electrolyte-gated devices. Currently, gold electrodes for WGFETs are mostly realized by means of photolithography. Inkjet printing of gold electrodes represents a more sustainable, fast, and cost-effective alternative to photolithography when nanometric resolution is not required. However, its use is limited by the high sintering temperatures of most of the available inks,^21^ which are not compatible with the low thermal budget of edible substrates.

With the aim to obtain inkjet printed electrodes on edible substrates for future edible transistors, here we have explored the use of an alternative Au nanoparticle formulation introduced by Kanehara *et al.*^22–24^ Ink nanoparticles possess a metal core surrounded by aromatic molecules acting as a conductive bridging ligand, and a conductive film can be obtained by low temperature drying without the need for actual sintering. Further advantages of this colloidal gold ink employed are its green water-based composition and stability in an ambient environment, besides the fact that it is commercially available (DryCure Au-J 1010B, C-INK Co., Ltd).

**Fig. 1:**
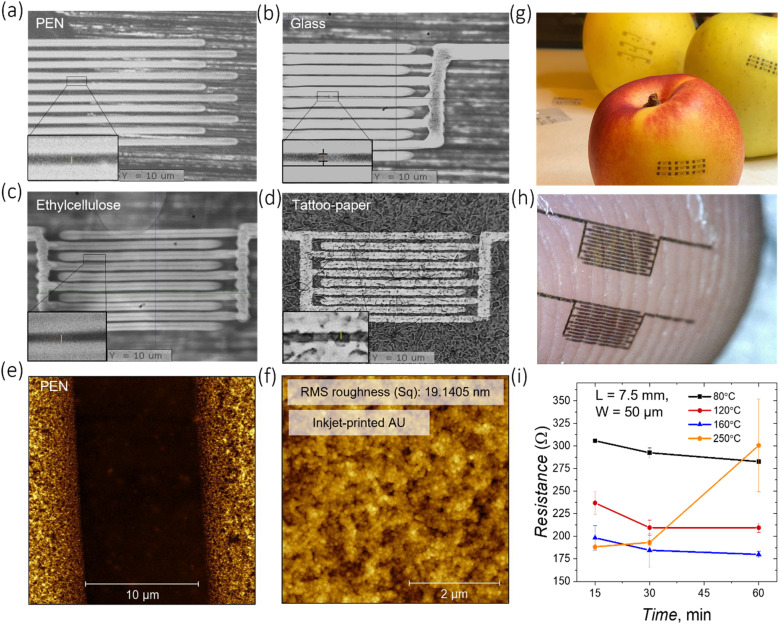
Structural, morphological, and electrical characterization of inkjet printed gold electrodes on different conventional and edible substrates. (a)–(d) Gold interdigitated electrodes inkjet printed on different substrates: PEN, glass, edible ethyl cellulose and tattoo paper substrates. (g) and (h) Digital photographs of gold electrodes transferred onto different surfaces: (top) a peach, an apple and (bottom) a fingertip. (e) AFM of gold electrodes inkjet printed on PEN: topography of the area between the two conducting fingers of interdigitated electrodes with a channel length of 10 μm; the area of the image is 20 × 20 μm^2^. (f) AFM topographic image of the top surface of the gold electrode. The image area is 5 × 5 μm^2^. (i) Dependence of the electrical resistance of inkjet printed gold lines under drying conditions. The plots are presented for lines with a length of 7.5 mm. Each point corresponds to an average value measured over 7 samples.

Prior to employing inkjet printed gold in devices, the optimization of the inkjet printing process (Fig. S1†) was carried out along with morphological, structural, and electrical analysis of printed gold traces. The results were then compared with electrodes patterned *via* a conventional lift-off photolithographic process. The printability of the gold ink has been assessed on conventional substrates, such as poly(ethylene 2,6-naphthalate) (PEN) and glass, and on edible ones, such as ethyl cellulose and tattoo paper (Fig. 1a–d). Apart from the state-of-the-art conventional glass substrate, PEN represents a common substrate for the development of flexible electronic devices. In particular, PEN finds an extensive use as a platform for inkjet printing,^25^ and thus, the optimization of gold patterning on PEN with competitive resolution and quality is relevant. Inkjet printing of gold electrodes on edible substrates, on the other hand, is particularly appealing for future realization of edible devices. Among numerous edible materials potentially suitable to be used as a substrate, the ethyl cellulose biopolymer (food additive E462) is of particular interest. Ethyl cellulose is a cellulose derivative and one of the few non-water-soluble biopolymers commonly used in food, cosmetic and pharmaceutical industries as a coating agent, flavouring fixative, binder, filler, thickener, film-former, drug carrier, or stabilizer.^26,27^ Thanks to their transparency, flexibility, and biocompatibility, ethyl cellulose substrates have been integrated into ingestible form factors, such as pills or capsules. However, recent research has gone beyond these traditional applications and explored the use of ethyl cellulose in the fabrication of advanced edible electronics. Specifically, it has been demonstrated that ethyl cellulose substrates can be used to produce edible supercapacitors and triboelectric nanogenerators.^28^ The preparation of the ethyl cellulose substrate used in the study is described in the Experimental section, and the appearance of the final substrate (thickness ≈30 μm) is shown in Fig. S2.† Tattoo paper as a substrate represents another appealing platform for edible electronics due to its ability to conformally adhere to different surfaces. Such a tattoo paper is composed of three layers: a paper carrier, a sacrificial starch/dextrin layer and a functional layer of ethyl cellulose (hundreds of nanometers thick).^29^

The printing parameters for the gold ink, used for each substrate, are reported in the ESI.† The drop spacing, *i.e.*, the distance between the centre of two contiguous inkjet printed droplets, was varied on different substrates in order to tune the thickness and the quality of the printed features and achieve an optimal droplet coalescence in printed lines, having a straight contour without line bulging. Inkjet printed interdigitated electrodes with remarkable resolution, in terms of both channel length and electrodes quality, were achieved on all the employed substrates, with critical features as small as 10 μm (Fig. 1e). The scalability of the electrode channel length on different substrates is demonstrated in Fig. S3, S4 and S5.† It is important to highlight that by employing tattoo paper as a substrate, printed electrodes and subsequent devices can be easily transferred and conformed to various objects, in particular, food items or skin, maintaining their integrity (Fig. 1g and h). To assess the relationship between electrical performances of inkjet printed gold traces and different drying conditions, traces with a length of 7.5 mm, a width of 50 μm and a thickness of 60 nm were printed on glass substrates and annealed using different combinations of temperature and time (Fig. 1i). The resistance values decrease when annealing the sample at a fixed temperature for a longer time, and the lower the temperature, the longer the time it takes to reach low resistance values. When increasing the annealing temperature from 80 °C to 250 °C, the resistivity decreases to (7.6 ± 0.5) × 10^−8^ Ω m at 160 °C and does not further significantly change. The obtained value of resistivity is in agreement with the values reported in the literature for gold inks^22^ and is of the same order of magnitude as that of bulk gold (2.2 × 10^−8^ Ω m). The resistance values linearly scale with the length of the printed traces, as expected (Fig. S11a†). A sharp increase of resistance was observed when annealing printed gold at 250 °C for 1 h, which can be explained by mechanical deformation of the traces (Fig. S11b and c†), possibly due to a thermal damage caused by high temperature. Annealing of the inkjet printed gold at 120 °C for 1 hour was found to be a suitable trade-off condition to provide a sufficient conductivity of the metal and to enable low-temperature processing on soft edible substrates.

A morphological characterization of the annealed gold electrodes was carried out by atomic force microscopy (AFM). The printed electrodes on PEN feature smooth edges (Fig. 1e). However, in few cases, and especially when using glass substrates, blurred edges with small gold clusters extending towards the channel region may be present (Fig. S8 and S9†). The top surface of the electrode has a dense nanostructured morphology with a root mean square (RMS) roughness of ≈19.1 nm (Fig. 1f). As a comparison, photolithographically-patterned gold is characterized by a smoother surface with a RMS roughness of ∼2.15 nm, one order of magnitude lower (Fig. S6a†). The printed Au nanoparticles have a spherical shape (size of ∼45 nm) that is observed throughout the printed traces (Fig. S6b†). The thickness of the gold electrodes on PEN has been evaluated at the edge of the inkjet-printed film and was found to be ∼45 nm (Fig. S7†). An equivalent morphological characterization of gold electrodes has been carried out when inkjet printed on corning glass substrates (Fig. S8 and S9†). AFM is not viable on edible substrates due to their flexibility and poor planarity, therefore scanning electron microscopy (SEM) was employed instead in the case of ethyl cellulose. SEM images of gold interdigitated electrodes with a channel length *L* = 10 μm are shown in Fig. S10.†

### 2.2 Fully printed water-gated transistors

The electrical performances and quality of inkjet printed gold electrodes were assessed by fabricating fully printed low-voltage WGFETs with PEN as reference test substrates. Examples of all inkjet printed WGFETs with gold electrodes with channel lengths in the order of few tens of microns have not been demonstrated in the literature until now.

**Fig. 2:**
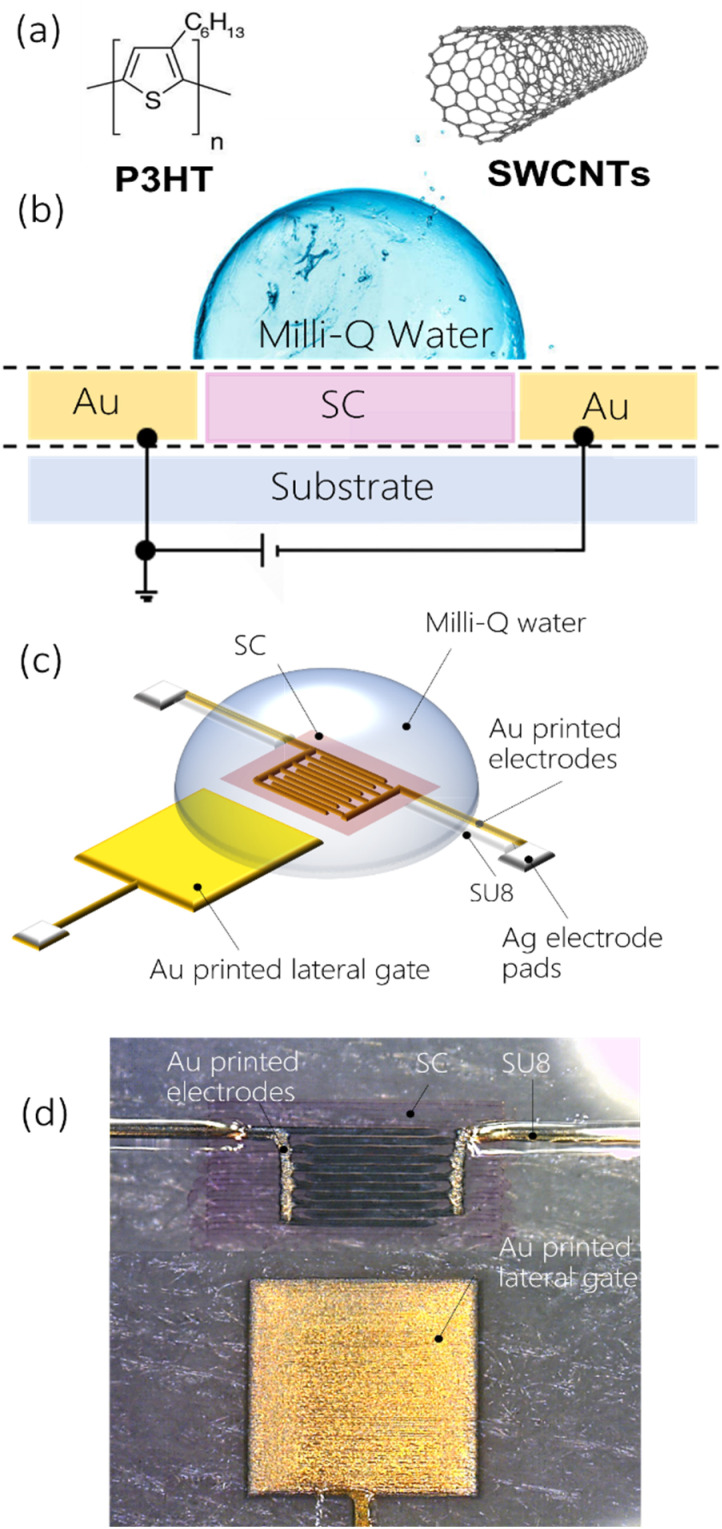
(a) Chemical structure of the semiconducting materials used (P3HT and SWCNTs). (b) Schematic cross-section and (c) representation of a fully printed WGFET. (d) Microscopy image of the device on a PEN substrate.

A schematic cross-section of the fabricated WGFETs, with a planar side-gate configuration, is shown in Fig. 2. By exploiting the good control and resolution obtained with the selected gold ink, source and drain interdigitated electrodes were inkjet printed to achieve a channel length *L* of 10 μm, with a channel width *W* of 10^5^ μm. Gold gate electrodes of a square shape, with 2.5 mm sides, were patterned using the same technique at 1 mm distance from the channel (Fig. S12†). Subsequently, to avoid the occurrence of parasitic currents between the contact lines and the gate electrode through the electrolyte, a UV-curable epoxy passivation layer of SU-8 was patterned by inkjet printing on top of all gold traces, except for the channel and the lateral gate. Two solution-processible organic semiconductors were used in our tests to demonstrate the compatibility of fully printed WGFETs with different active materials. Specifically, a regioregular poly(3-hexylthiophene) (P3HT) polymer and polymer-wrapped semiconducting (6,5) single-walled carbon nanotubes (s-SWCNTs) were employed; they are printable, they offer advantages in terms of electronic properties and stable operation in water, as previously reported in the literature.^16,30^ In addition, both materials already found applications in bioelectronics.^31–33^ P3HT and s-SWCNT semiconductors were inkjet printed into the channel region, and a drop of pure water (Milli-Q, Millipore) was then cast on the semiconductor surface under ambient conditions to fully cover the gate terminal and the channel regions. Nominally identical devices, but with photolithographically patterned gold electrodes, were fabricated and used as a benchmark to assess the functional performances of the fully inkjet printed WGFETs.

**Fig. 3:**
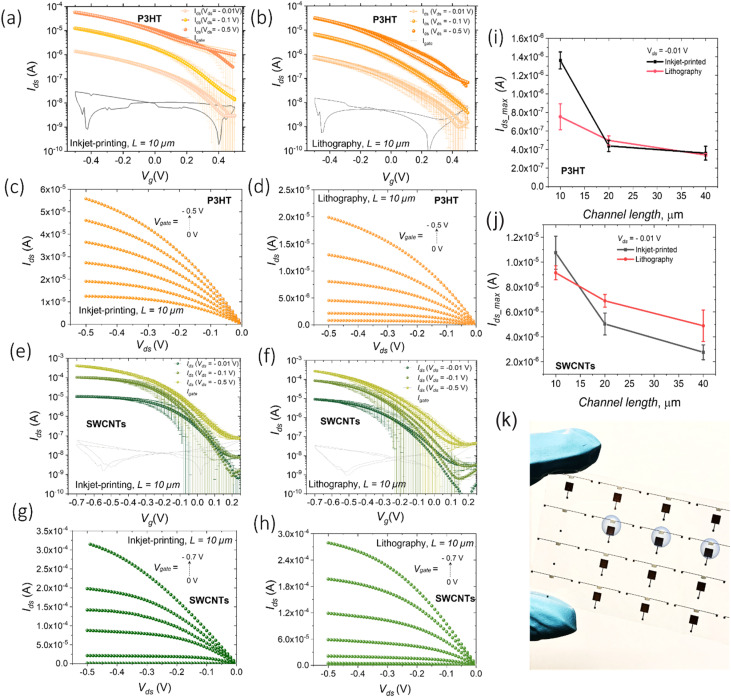
Electrical characterization of WGFETs. (a) Transfer and (c) output characteristic curves of P3HT-based WGFETs with inkjet printed gold electrodes, and (b) transfer and (d) output curves of devices with photolithographically patterned gold electrodes in linear (*V*_ds_ = −0.01 V; −0.1 V) and saturation (*V*_ds_ = −0.5 V) regimes; the *V*_gate_ sweep rate was 10 mV s^−1^; transfer curves are averaged over 7 samples; geometrical parameters of WGFETs: *L* = 10 μm; *W* = 10^5^ μm. (i) Comparison of max *I*_ds_ values for two different configurations of P3HT-based WGFETs (based on inkjet printed and photolithographically patterned gold electrodes) in the linear regime (*V*_ds_ = −0.01 V) for devices with different channel lengths: *L* = 10 μm, *L* = 20 μm, and *L* = 40 μm. (e) Transfer and (g) output characteristic curves of s-SWCNT-based WGFETs, with inkjet printed gold electrodes, and (f) transfer and (h) output curves for devices with photolithographically patterned gold electrodes in linear (*V*_ds_ = −0.01 V; −0.1 V) and saturation (*V*_ds_ = −0.5 V) regimes; the *V*_gate_ sweep rate was 10 mV s^−1^; transfer curves are averaged over 7 samples; geometrical parameters of OFETs: *L* = 10 μm; *W* = 10^5^ μm. (j) Comparison of max *I*_ds_ values for two different configurations of s-SWCNT-based WGFETs (based on inkjet printed and photolithographically patterned gold electrodes) in the linear regime (*V*_ds_ = −0.01 V) for the devices with different channel lengths: *L* = 10 μm, *L* = 20 μm, and *L* = 40 μm. (k) A digital image of WGFETs.

The current–voltage (*I*–*V*) transfer and output characteristics of the P3HT-based devices are given in Fig. 3a–d. A typical p-type field-effect transistor operation in both linear (*V*_ds_ = −0.01 V; *V*_ds_ = −0.1 V) and saturation regimes (*V*_ds_ = −0.5 V) at gate voltages *V*_g_ between 0.5 V and −0.5 V is demonstrated for both device configurations. The threshold voltage *V*_th_ was found to be ≈0.4 V for WGFETs with inkjet printed electrodes and ≈0.34 V for devices with photolithographically patterned electrodes at *V*_ds_ = −0.5 V. The transfer voltage sweeps exhibit a small hysteresis and a more evident *V*_ds_ dependent shift of *V*_th_ in both cases, which might be related to field-dependent charge injection.^34^ The maximum currents achieved at *V*_g_ = −0.5 V and different applied *V*_ds_ lie within the same order of magnitude for both the configurations of devices, also for longer channel lengths (*L* = 20 μm and *L* = 40 μm, Fig. S13 and S14†). Fig. 3i shows a comparison of maximum *I*_ds_ for two different configurations of P3HT-based WGFETs in the linear regime (*V*_ds_ = −0.01 V) for the devices with different channel lengths. WGFETs with *L* = 10 μm, *i.e.*, the smallest channel length employed, and inkjet printed electrodes feature slightly higher currents (*I*_d_ = 1.4 × 10^−6^ A, *V*_ds_ = −0.01 V) in comparison with the devices with photolithographically patterned electrodes with the same nominal channel length (*I*_d_ = 7.1 × 10^−7^ A, *V*_ds_ = −0.01 V). This can be likely related to improved charge injection provided by gold inkjet printed electrodes^22^ or a better semiconductor film quality/morphology on top of printed electrodes. Nevertheless, the blurred edge of printed electrodes, with gold clusters, may contribute to the improved device performances. Further investigations will clarify this aspect.

The values of the mobility–capacitance product *μ*_sat_·*C* have been extracted for P3HT-based WGFETs with *L* = 10 μm from the transfer characteristic curves in the saturation regime. The determined *μ*_sat_·*C* values are ∼0.16 μF V^−1^ s^−1^ for WGFETs with inkjet printed electrodes and ∼0.13 μF V^−1^ s^−1^ for the configuration with photolithographically patterned electrodes. These values indicate how fully printed devices can achieve performances matching transistors realized using conventional photolithographic techniques.

The stabilization of the proposed WGFETs based on inkjet printed gold electrodes when operating in water, important for any use in biosensing applications, was evaluated by measuring a series of consecutive transfer characteristic curves acquired with 30 min intervals for a duration of 14 h (Fig. S15†). The device behaviour is typical for P3HT-based WGFETs, as previously reported in the literature.^30,35^ It can be seen that a sudden decrease in the current occurs in the first few cycles, whereas the current level almost reaches a steady state after 14 hours.

A similar comparison was drawn for WGFETs fabricated by employing s-SWCNTs as an active material (Fig. 3e–h). In this case, the drain current values for printed and non-printed electrodes are very similar also for 10 μm channel devices. This indicates that the reproducible increase with P3HT devices is likely related to injection issues when the polymer is adopted.

The determined *μ*_sat_·*C* values for s-SWCNT-based WGFETs were found to be ∼2.5 μF V^−1^ s^−1^ for the configuration with inkjet-printed electrodes and ∼2.0 μF V^−1^ s^−1^ for the configuration with photolithographically patterned electrodes. Again, the performances are almost identical to fully inkjet printed transistors and devices employing photolithographically patterned electrodes.

The data reported here clearly show how the inkjet printed gold electrodes are qualified for use in edible electronics, as well as for other areas of research, such as organic bioelectronics, where WGFETs are the core for state-of-the-art biosensing platforms.^36^

### 2.3. Chitosan-gated organic transistors and circuits operating in air

**Fig. 4:**
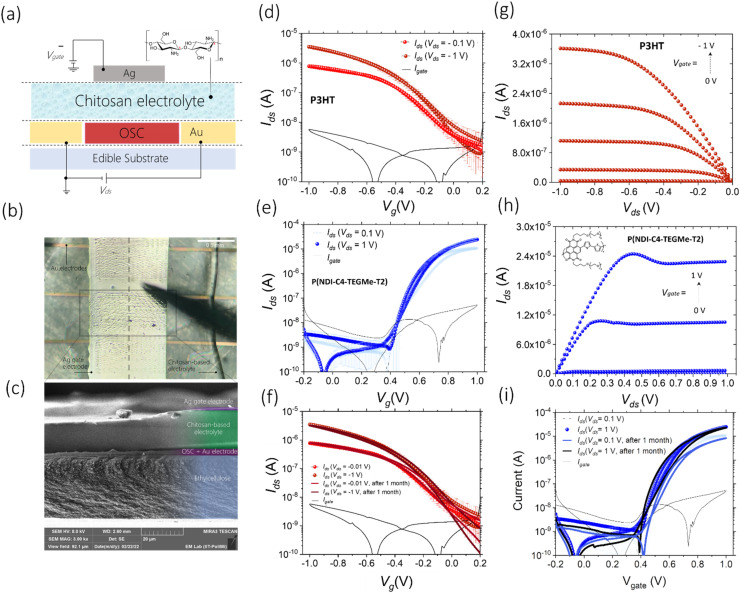
Structure, fabrication, and electrical characterization of fully printed chitosan-gated transistors on an edible substrate. (a) Schematic structural representation of the device. Chemical structure of chitosan is shown in the inset. (b) Microscopy image of three fully printed chitosan-gated transistors on an ethylcellulose substrate with a common silver gate electrode (top view). The dashed line indicates the cross-section imaged *via* SEM (c). It is possible to distinguish the layers composing the device: an ethylcellulose substrate (30 μm), a layer of a semiconductor deposited on the gold source and drain electrodes, a chitosan-based electrolyte (15 μm), and a silver gate electrode. The layer of the semiconducting polymer was printed approximately 10 times thicker than in the actual device to help distinguish it in the stack with other layers. (d) Transfer and (e) output characteristic curves of fully printed p-type chitosan-gated transistors on an ethylcellulose substrate in linear (*V*_ds_ = −0.1 V) and saturation (*V*_ds_ = −1 V) regimes (average curve over 7 samples); the *V*_gate_ sweep rate was 5 mV s^−1^. (f) Transfer and (g) output characteristic curves of fully printed n-type chitosan-gated transistors on an ethylcellulose substrate in linear (*V*_ds_ = 0.1 V) and saturation (*V*_ds_ = 1 V) regimes (average curve over 7 samples); the *V*_gate_ sweep rate was 2 mV s^−1^. Shelf-life stability of fully printed (h) p-type and (i) n-type chitosan-gated transistors. A comparison between electronic performances (transfer characteristics in linear and saturation regimes) after a 1-month control period of storing the devices in air is shown.

Herein, we assess the performances of a low-cost, widely accessible, edible and nutritive polysaccharide, chitosan, as the base of a solid-state electrolytic gate dielectric for the realization of a platform for edible electrolyte gated FETs, in combination with inkjet-printed electrodes and edible substrates. Chitosan is mainly obtained from shrimp and crab shells, and owing to its excellent antimicrobial, biocompatible, and biodegradable properties, it is actively used as a food additive and coating, a dietary supplement and a drug delivery system.^37–39^ We have blended chitosan with glycerol (20% w/w), which is also recognized as an edible food additive (E422), and has the function of a plasticiser to improve the mechanical properties and to induce a more amorphous-like phase in the film, with a consequent increase in the ionic conductance. By drop-casting 10 μL of this blend solution on an ethyl cellulose substrate, thicknesses ranging between 30 and 40 μm are achieved. Electrochemical impedance spectroscopy (EIS) was performed on a metal–insulator–metal architecture to characterize the chitosan blend properties as an electrolyte, and the results demonstrate that the formation of EDLs at the interface with metallic electrodes does not depend on the electrolyte thickness and produce a capacitance of ≈8 μF cm^−2^ (Fig. S21†). The schematic structure of the fully printed chitosan-gated transistors, employing inkjet printed bottom source–drain gold electrodes and an inkjet printed Ag top gate, is shown in Fig. 4a and b. We used silver as a cheaper alternative to gold for the printed gate, as its simple geometry does not require a very fine accuracy. Given the lack of approved edible semiconductors, two model polymers were used in the following tests to demonstrate the compatibility of chitosan-gated transistors with both hole and electron transporting materials. Biocompatible P3HT,^40–42^ used in the previous section, was employed as a reference hole transporting polymer semiconductor for p-type chitosan-gated transistors. P(NDI-C4-TEGMe-T2), a modified version of P(NDI2OD-T2) bearing polar triethylene glycol-based (TEG-based) side chains, was used as an electron transporting material in n-type devices. The cytotoxicity of the P(NDI-C4-TEGMe-T2) polymer has been evaluated through tests on cell proliferation and specifically on MDCK-II and HEK-29 cell lines, with no adverse effects observed (Fig. S19†). Both P3HT and P(NDI-C4-TEGMe-T2) polymers were inkjet-printed onto the substrates provided with interdigitated contacts, while the chitosan-based electrolyte was simply drop-cast on the device channel under ambient conditions. A SEM cross-section image of the device stack is shown in Fig. 4c.

The *I*–*V* transfer and output characteristics of the p-type and n-type devices are presented in Fig. 4d, g and e, h. The curves demonstrate a typical transistor operation in both linear (*V*_ds_ = ±0.1 V) and saturation regimes (*V*_ds_ = ±1 V) at absolute gate voltages, *V*_gate_, below 1 V. It can be observed that a kink develops in the output curves of P(NDI-C4-TEGMe-T2) devices with increasing gate bias. We ascribe this non-ideality to bias stress effects, more evident at a higher charge density. The threshold voltage *V*_th_ was found to be ≈−0.13 V for P3HT-based chitosan-gated transistors and ≈0.5 V for P(NDI-C4-TEGMe-T2)-based chitosan-gated transistors at *V*_ds_ = ±1 V. A distinctive feature of the demonstrated chitosan-gated transistors is their reproducibility (the data are averaged over an array of 7 devices), negligible hysteresis and stable operation in air at room temperature (relative humidity of about 50%).

The extracted *μ*_sat_·*C* values in the saturation regime are ≈0.0082 μF V^−1^ s^−1^ for p-type and ≈0.274 μF V^−1^ s^−1^ for n-type transistors. A marked difference in such values can be explained by the fact that P(NDI-C4-TEGMe-T2)-based n-type devices operate as organic electrochemical transistors (OECT), as was demonstrated in a previous work,^43^ and feature, therefore, a volumetric capacitance that increases with increasing semiconductor thickness up to hundreds of μF cm^−2^.^44^ Instead, it is well known that electrolyte-gated P3HT devices rely on the formation of electrical double layers at the semiconductor–electrolyte interface, producing a lower capacitance of the order of few μF cm^−2^.^17,45^

The operational stability of the p-type and n-type chitosan-gated transistors has been assessed through a continuous cycling voltage test performed in air by switching the devices on and off every 40 s (30 min total duration, Fig. S18†). Both types of devices revealed stable operation, and no evidence of electronic performance degradation was observed after 40 cycles. In addition, the devices demonstrated a shelf-life stability up to one month, while being stored in air (Fig. 4f and i).

**Fig. 5:**
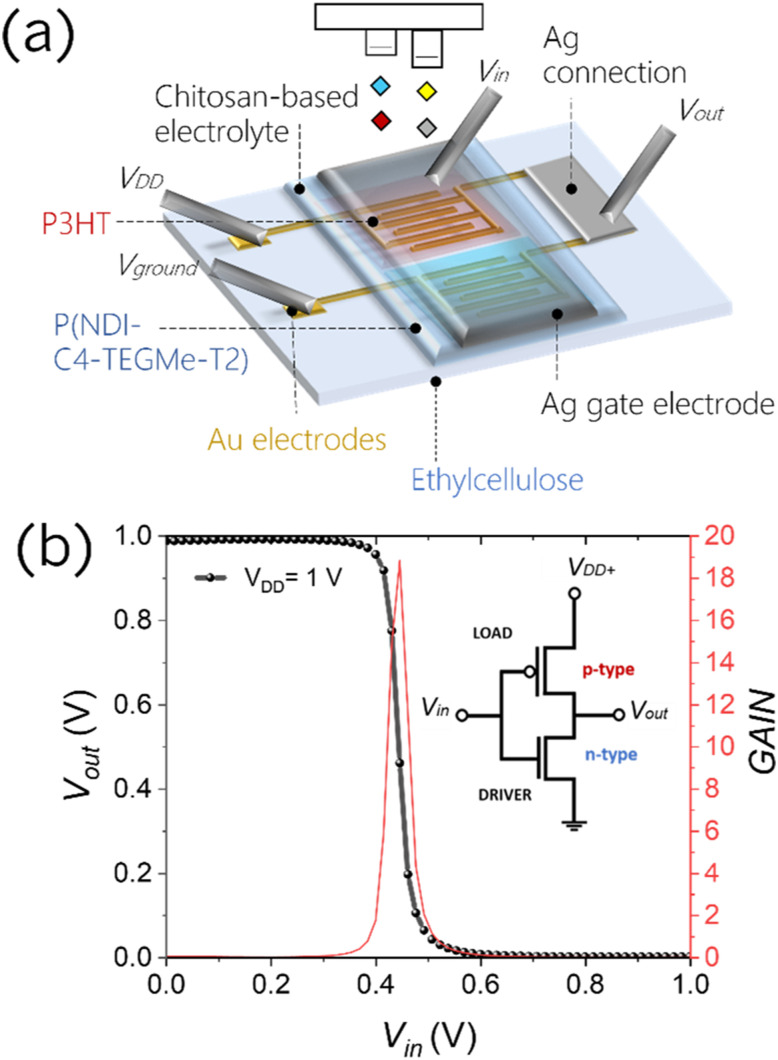
(a) Schematic representation of the inverting logic gate device realized with chitosan-gated transistors based on P3HT and P(NDI-C4-TEGMe-T2); *L* = 10 μm and *W* = 10 000 μm for the p-type device and *L* = 30 μm and *W* = 10 000 μm for the n-type device. (b) Complementary inverter voltage transfer curve (VTC) and the corresponding derivative curve to extract gain as a function of input voltages; the sweep rate is 15 mV s^−1^; the inverter configuration is given as an inset.

The possibility to realize both p-type and n-type devices allows for the realization of complementary logic circuits gated through chitosan. Here we fabricated an inverter based on chitosan-gated transistors on an ethyl cellulose substrate. Fig. 5a shows a visual representation of the device. The resulting logic gate shows a voltage transfer characteristic (VTC) with an inversion threshold (*V*_inv_) of ≈0.45 V at *V*_DD_ = 1 V, close to the ideal *V*_DD_/2 value, which could be achieved by further optimization of the geometrical parameters of single transistors. The inverter shows a high gain of ≈−19 at the logic transition.

The transistors integrated in the inverter employ to a great extent edible materials approved by the FDA or potentially edible materials in their design. Estimated amounts of materials constituting a single inverter are reported in Table 1 in grams per device. The ethyl cellulose substrate, a food additive, E462, approved by the FDA does not pose any safety concerns, as it can be ingested even at significant quantities of ≈660–900 mg kg^−1^ daily.^46^ Both gold and silver are commonly used in their bulk form as food garnishment, and are listed as food additives, E175 and E174, correspondingly, with reference daily intakes of few μg kg^−1^.^20,47^ However, the metallic films realized here are formed starting from nanoparticle ink formulations. Therefore, they cannot be placed within the same FDA categorization,^48^ and shall be the subject of future toxicology investigations. The limited quantities of gold and silver used here for a single device, estimated to be ≈2 μg and ≈10 μg, respectively, will clearly play an important role in the final toxicology profile of a circuit. On the other hand, chitosan and glycerol are nutritious edible compounds that find a wide application not only in the food sector but also in medicine, with intakes as high as 6 g day^−1^ and 2 g kg^−1^ day^−1^, correspondingly. The quantities of chitosan and glycerol in the final device are significantly below the stated values, and amount to 0.2 mg and 0.04 mg, respectively. As far as the semiconducting materials are concerned, neither P3HT or P(NDI-C4-TEGMe-T2) have been tested for edibility. However, P3HT has been identified as biocompatible and used in a number of studies concerning cell proliferation, while P(NDI-C4-TEGMe-T2) was successfully subjected to cytotoxicity tests on cell lines (Fig. S19†). Furthermore, deposition *via* inkjet printing of both semiconductors allows to use extremely low quantities in the range of picograms per device. In any case, the performances of the reported devices show how it is possible to build a potentially edible platform for the benchmarking of edible semiconductors, *e.g.* carotenoids.^49^

**Table 1:** Estimated amounts of materials constituting a single inverter based on chitosan-gated transistors in grams per device, with the corresponding reported daily intake and FDA *E* value

Material	Dose per device	Allowed daily intake
P3HT	4 pg	N.A.
P(NDI-C4-TEGMe-T2)	4 pg	N.A.
Ethylcellulose	∼3 mg	660–900 mg kg^−1^ day^−1^ (E462)
Printed gold	2 μg	N.A. (1.32 μg kg^−1^ day^−1^ for E175 edible gold)
Printed silver	10 μg	N.A. (12 μg kg^−1^ day^−1^ for E174 edible silver)
Chitosan	0.2 mg	6 g day^−1^
Glycerol	0.04 mg	2 g kg^−1^ day^−1^ (E422)

Finally, as a proof of concept for a possible future application, we take advantage of the flexibility of the ethyl cellulose substrate to show how it is possible to fabricate foldable electronic devices that can be integrated inside edible (gelatine) pharmaceutical capsules, commonly used for drug delivery (Fig. S20†). Further tests are required to fully assess the viability of this strategy, which could open the way to a new plethora of applications, like *in situ*, and real time monitoring of drug delivery through safe, edible and digestible materials and devices.^6^

## 3. Conclusion

In this work, we presented a platform comprising both materials and processing conditions for the realization of printed and potentially edible transistors operating at low voltage. First, we report the successful inkjet printing of a water-based gold ink on conventional and edible substrates, achieving critical lateral features as low as 10 μm. Fully printed WGFETs were then realized on PEN substrates and benchmarked against identical devices with contacts patterned by photolithography. The comparative analysis of the two configurations revealed very similar performances in terms of the mobility–capacitance product, on/off ratio, and negligible hysteresis. This result shows how high-performance fully inkjet printed devices can be realized in a reproducible manner, substituting photolithography when nanometric resolution is not required, by providing an additive, faster, cheaper and solution-processable alternative.

Next, we have presented the use of chitosan, an edible and inexpensive polysaccharide, as the base of a solid electrolyte dielectric realized through blending with edible glycerol, for the realization of edible transistors. Printed chitosan-gated complementary n- and p-type transistors and logic circuits (namely inverting logic gates), operating at low voltages (<1 V), were demonstrated on flexible edible ethyl cellulose substrates. The devices exhibit promising electronic performances in terms of the mobility–capacitance product and on–off current ratio, operation stability in air, and a shelf-life of up to 1 month. Flexibility and small dimensions of the devices allow for their easy integration in edible carriers, such as pharmaceutical capsules.

The edible substrate and gating medium represent together more than 99% weight fraction of the total dose of materials per single logic inverter. The residual fraction, less than 1% of the total, is due to the printed inert metals and semiconductors, whose edibility has not been assessed yet, present only in traces, *i.e.* picograms per transistor. Ongoing research on edible semiconductors will provide active components made of edible dyes. The platform we have reported for edible transistors and circuits will allow on the one hand the benchmarking of such edible semiconductors, and on the other hand the development of circuits and biosensors safe to ingest, contributing to the development of future edible electronic systems for healthcare and food safety.

## 4. Experimental section

### 4.1. Materials

The gold ink, DryCure Au-J 1010B (10 cps, 10 wt%), was obtained from C-INK Co., Ltd, and used as it is, being stored in an ambient environment. The poly(ethylene 2,6-naphthalate) (PEN) substrate (125 μm thick) was purchased from Du Pont. Commercial tattoo paper (Tattoo 2.1) was obtained from The Magic Touch Ltd. Ethylcellulose powder (48.0–49.5% (w/w) ethoxyl basis) was provided by Sigma Aldrich. Regioregular P3HT (regioregularity >99%; MW = 17.5 kDa) was purchased from Sigma-Aldrich and used as received without any further purification. s-SWCNTs were extracted from CoMoCAT raw carbon nanotubes (Chasm Advanced Materials, SG65i-L58) *via* selective polymer wrapping with poly[(9,9-dioctylfluorenyl-2,7-diyl)-*alt*-(6,6′-(2,2′-bipyridine))] (PFO-BPy, American Dye Source, M_W = 40 kg mol^−1^). p(NDI-C4-TEGMe-T2) was synthetized according to the procedure reported elsewhere.^43,50^ Chitosan (low molecular weight), glycerol and the AgNP ink (Silverjet DGP-40LT-15C) were purchased from Sigma Aldrich.

### 4.2. Preparation of the ethyl cellulose substrate

Ethylcellulose films used as substrates were prepared following the method described by Lamanna *et al*.^28^ A 2% (w/v) solution of ethylcellulose in ethanol was prepared by constant stirring of the solution for 1 hour using a magnetic stirrer at room temperature. After the formation of a homogenized solution, 20 ml of the solution were gently poured into a Petri dish (diameter of 15 cm) avoiding any bubble formation. The content of the Petri dish was then dried overnight in an oven at 50 °C, resulting in a film that was manually peeled unbroken from the casting surface using water. The flat side of the film, which was in contact with the Petri dish, was employed as a working surface for inkjet printing.

### 4.3. Fabrication of WGFETs with photolithographically patterned gold electrodes

To fabricate WGFETs, we adopted a planar side-gate configuration. The gold source (S), drain (D) and square lateral gate electrodes (50 nm thick) were defined by a lift-off photolithographic process on the PEN substrate (*W* = 10 000 μm; channel lengths of 10, 20 and 40 μm; lateral gate with a side of 2.5 mm). With this technique, a single photoresist layer was obtained (AZ5214E, MicroChem). It was first spin coated on the substrate (1 min at 4000 rpm), then baked at 110 °C for 90 s in order to remove the solvent, and subjected to a negative exposition using a maskless aligner (Heidelberg MLA100). Cross-linking of the exposed parts of the resist was then carried out by baking the sample at 120 °C for 90 s. The following flood exposure of the whole sample allows to obtain the desired pattern after the development in an AZ 726 MIF developer. Before the thermal evaporation of gold (48 nm), an adhesion layer of chromium (2 nm) was deposited over the PEN substrate. The lift-off process was carried out overnight by immersion of the sample in a TechniStrip Microdeposit D2 stripper. Finally, the samples were cleaned in an ultrasonic bath in both acetone and IPA, dried with nitrogen and further treated with oxygen plasma for 2 min. Furthermore, using a Fujifilm Dimatix DMP-2831 printer, inkjet-printing deposition of the remaining layers was carried out with an adopted Samba cartridge, which provides a drop volume of 2.4 pL. In order to reduce the leakage current, a film of a biocompatible insulator (SU8 – TF6001 MicroChem) was inkjet-printed on top of the gold traces to be exposed to the electrolyte, with the exception of the channel and the lateral gate areas. A drop spacing of 20 μm, a firing voltage of 34 V, and a jetting frequency of 5 kHz were used. The devices were than annealed at 130 °C for 30 min and UV cured in an inert atmosphere.

Next, a P3HT solution and a s-SWCNT ink were prepared. P3HT was dissolved in a mixture of chlorobenzene (CB) and *ortho*-dichlorobenzene (ODCB), in a ratio of 3 : 1 v/v, at a concentration of 2.6 mg mL^−1^ and inkjet-printed over the channel area of the device, previously treated with oxygen plasma for 3 min. A drop spacing of 45 μm, a firing voltage of 40 V, a jetting frequency of 1 kHz and a substrate temperature of 35 °C were used for deposition. The devices were finally annealed at 130 °C in an inert atmosphere for 1 h.

A (6,5) s-SWCNT dispersion was prepared from CoMoCAT carbon nanotubes (Chasm Advanced Materials, SG65i-L58) following a previously reported protocol.^16^ The nanotube ink was prepared through redispersion of the s-SWCNT filter cake in a mixture of ODCB and CB, in a ratio 2 : 1 v/v, by 30 min of bath sonication, and an optical density of 1 cm^−1^ was obtained at the E_11_ transition. The ink of s-SWCNTs was then inkjet-printed over the channel area of the device, previously treated with oxygen plasma for 3 min. 9 layers were deposited with a drop spacing of 60 μm, a firing voltage of 40 V, and a jetting frequency of 1 kHz, and a substrate temperature set to 20 °C. The s-SWCNT films were finally annealed at 120 °C in air for 20 min and rinsed with THF and IPA for excess polymer removal.

As a final step, a drop of ultrapure (Milli-Q, Millipore) water was poured onto the sample to fully cover the gate electrode and the channel regions.

### 4.4. Fabrication of WGFETs with inkjet printed gold electrodes

WGFETs were prepared with a planar side-gate configuration through a process identical to the one described above, except for the gold source (S), drain (D) and square lateral gate electrodes, which were patterned by inkjet printing on the PEN substrate. The gold NP ink was deposited on the PEN substrate with a drop spacing of 20 μm and a jetting voltage 37 V, and a substrate temperature set to 60 °C. Prior the deposition of the square lateral gate, the samples were exposed to 20% oxygen plasma for 1 min. The gold was sintered afterwards at 120 c for 1 hour.

### 4.5. Preparation of the chitosan-based electrolyte

A chitosan-based electrolyte was prepared by dissolution of chitosan in 1% acetic acid aqueous solution, at a concentration of 7.5 g l^−1^, by stirring the solution at room temperature overnight. Glycerol (food additive E422) was then added, in a quantity equal to 20% of the chitosan weight. The resulting solution was further stirred for 20 min at room temperature and stored at 4 °C.

### 4.6. Fabrication of fully printed chitosan-gated transistors

The ethyl cellulose substrate was fixed on a glass slide with a PDMS thin layer to provide a flat robust surface for inkjet printing of the following layers. Before the deposition of gold electrodes, the substrate was treated with oxygen plasma for 1 min at 20% power. The inkjet printing deposition of the remaining layers was carried out using a Fujifilm Dimatix DMP-2831 printer with a Samba cartridge. The interdigitated Au source and drain contacts with *L* = 10 μm and *W* = 10^5^ μm were defined by inkjet printing with a drop spacing of 20 μm and the voltage applied to the piezoelectric transducer set to 37 V. The substrate was maintained at 60 °C during the printing process, and the electrodes were subsequently sintered at 120 °C in air for 20 min. P3HT was dissolved in ODCB at a concentration of 3 mg mL^−1^ and P(NDI-C4-TEGMe-T2) was dissolved in a mixture of CB and ODCB, in a ratio of 75/25 vol/vol, at a concentration of 2 mg mL^−1^ by stirring the solutions at ≈80 °C and at room temperature, respectively. The inks were stirred at the corresponding temperature for 20 min before deposition. P3HT was inkjet-printed over the channel area of the device with a drop spacing of 25 μm, a firing voltage of 29 V, and at a substrate temperature set to 35 °C. For the deposition of P(NDI-C4-TEGMe-T2), a drop spacing of 15 μm, a firing voltage of 30 V, and a substrate temperature set to 35 °C were used. The devices were subsequently annealed at 125 °C for 15 min in air to remove the residual solvent. Furthermore, the chitosan-based electrolyte was drop-cast on top of the semiconductor to cover the channel area and annealed at 80 °C for 30 minutes. Silver gates were inkjet printed as the last step on top of the solid electrolyte with a drop spacing of 40 μm and a substrate temperature set to 35 °C. The devices were finally sintered for 15 minutes at 100 °C and were easily peeled off from the glass slide using a tweezer. Chitosan-gated transistors of an identical architecture were realized on edible tattoo paper substrates following the same procedure.

### 4.7. Film and device characterization

The surface topography of the Au films was measured with an Agilent 5500 atomic force microscope operated in the acoustic mode. Cross-sectional characterization of selected samples was performed *via* field emission scanning electron microscopy (SEM Tescan MIRA III, an acceleration voltage of 8 kV).

To obtain a cross-section of the chitosan-gated transistors for SEM analysis, the devices were frozen in liquid nitrogen and subsequently fractured. A thin Au film (≈20 nm) was deposited over a sample by means of an Anatech Hummer 6.2 sputter coater to provide a conductive surface. In order to highlight different layers, SEM images were post-processed (colorized) with Adobe Photoshop.

The mass of all inkjet-printed materials was evaluated by considering the total number of droplets deposited (whose volume is known) and the concentration of the ink. For the evaluation of glycerol, ethyl cellulose and chitosan, a glass substrate was weighed with a precision scale before and after the deposition of these materials on top of it, and the difference was taken to be their mass. Both the substrates and the electrolyte were deposited in the same amount and conditions used for the realization of the devices.

Measurements of the resistance of gold traces and transfer and output characteristics of the devices were performed in air using an Agilent B1500A semiconductor parameter analyzer. All the devices were measured in a “yellow” room, *i.e.*, under light filtered below 500 nm. The devices were periodically exposed to a white-light microscope in order to probe the sample. Mobility–capacitance product values and threshold voltages were obtained by linear fitting of the square-root of *I*_ds_ in the saturation regime according to the following equation:
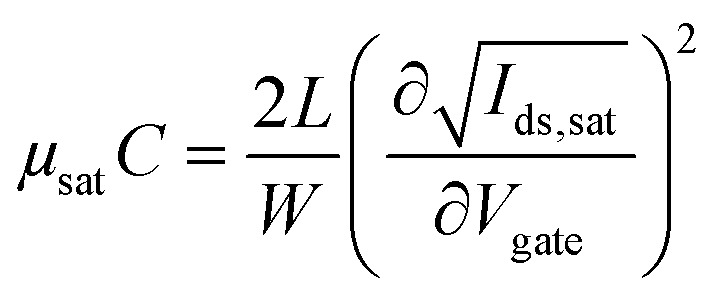
where *μ*_sat_ is the mobility in the saturation regime, *C* is the gate capacitance per unit area, *I*_ds_ is the drain current, and *V*_gate_ is the gate voltage.

## Conflicts of interest

The authors declare no conflict of interest.

## Supplementary Material

NR-015-D3NR01051A-s001
