# Correction: Chitosan-gated organic transistors printed on ethyl cellulose as a versatile platform for edible electronics and bioelectronics

**DOI:** 10.1039/d4nr90150a

**Published:** 2024-08-14

**Authors:** Alina S. Sharova, Francesco Modena, Alessandro Luzio, Filippo Melloni, Pietro Cataldi, Fabrizio Viola, Leonardo Lamanna, Nicolas F. Zorn, Mauro Sassi, Carlotta Ronchi, Jana Zaumseil, Luca Beverina, Maria Rosa Antognazza, Mario Caironi

**Affiliations:** a Center for Nano Science and Technology, Istituto Italiano di Tecnologia Via Raffaele Rubattino 81 20134 Milano Italy mario.caironi@iit.it; b Department of Physics, Politecnico di Milano Piazza Leonardo da Vinci 32 20133 Milano Italy; c Department of Electronics, Information and Bioengineering, Politecnico di Milano Piazza Leonardo da Vinci 32 20133 Milano Italy; d Smart Materials, Istituto Italiano di Tecnologia Via Morego 30 16163 Genova Italy; e Institute for Physical Chemistry, Heidelberg University 69120 Heidelberg Germany; f Department of Materials Science, Università degli Studi di Milano-Bicocca via Cozzi 55 20125 Milano Italy; g Department of Engineering for Innovation, University of Salento Via per Monteroni 73100 Lecce Italy

## Abstract

Correction for ‘Chitosan-gated organic transistors printed on ethyl cellulose as a versatile platform for edible electronics and bioelectronics’ by Alina S. Sharova *et al.*, *Nanoscale*, 2023, **15**, 10808–10819, https://doi.org/10.1039/D3NR01051A.

The authors regret that in the original article the weight of semiconductors present for each inverter was erroneously reported, due to a calculation error. Table 1 indicated a “Dose per device” of 4 pg for both P3HT and P(NDI-C4-TEGMe-T2), however, the correct doses are 8.4 ng for P3HT and 16 ng for P(NDI-C4-TEGMe-T2). The authors confirm that the main findings and conclusions of the work are not affected by this error. While the actual dose of semiconductors per inverter is three orders of magnitude greater than what erroneously reported in the original manuscript, such a value still represents a very small trace with respect to the total volume of the device, *i.e.*, nanograms *vs.* milligrams.

The correct Table 1 is reported below:

**Table 1:** Estimated amounts of materials constituting a single inverter based on chitosan-gated transistors in grams per device, with the corresponding reported daily intake and FDA *E* value

Material	Dose per device	Allowed daily intake
P3HT	8.4 ng	N.A.
P(NDI-C4-TEGMe-T2)	16 ng	N.A.
Ethylcellulose	∼3 mg	660–900 mg kg^−1^ day^−1^ (E462)
Printed gold	2 μg	N.A. (1.32 μg kg^−1^ day^−1^ for E175 edible gold)
Printed silver	10 μg	N.A. (12 μg kg^−1^ day^−1^ for E174 edible silver)
Chitosan	0.2 mg	6 g day^−1^
Glycerol	0.04 mg	2 g kg^−1^ day^−1^ (E422)

In addition:

- In the abstract: “including biocompatible polymers present in the picogram range per device” should be: “including biocompatible polymers present in the nanogram range per device”.

- On page 10816, left column, line 24: “range of picograms per device” should be: “range of nanograms per device”.

- On page 10816, right column, line 21: “*i.e.* picograms per transistor” should be: “*i.e.* nanograms per transistor”.

The Royal Society of Chemistry apologises for these errors and any consequent inconvenience to authors and readers.

