# Supplementary material for: Chitosan-gated organic transistors printed on ethyl cellulose as a versatile platform for edible electronics and bioelectronics
Source: Nanoscale. 2023 May 22;15(25):10808–19. doi: 10.1039/d3nr01051a (PMC10311466; doi:10.1039/d3nr01051a)
Supplement: NR-015-D3NR01051A-s001 [file NR-015-D3NR01051A-s001.pdf]

Electronic Supplementary Material (ESI) for Nanoscale.
This journal is © The Royal Society of Chemistry 2023

Supporting Information

Chitosan gated organic transistors printed on ethyl cellulose as a versatile platform for edible electronics and bioelectronics

Alina S. Sharova[a,b]‡, Francesco Modena[a,c]‡, Alessandro Luzio[a], Filippo Melloni[a,c], Pietro Cataldi[a,d], Fabrizio Viola[a], Leonardo Lamanna[a,g], Nicolas F. Zorn[e], Mauro Sassi[f], Carlotta Ronchi[a], Jana Zaumseil[e], Luca Beverina[f], Maria Rosa Antognazza[a] and Mario Caironi*[a]

a) Center for Nano Science and Technology @PoliMi, Istituto Italiano di Tecnologia, Via Raffaele Rubattino, 81, 20134 Milano, Italy.
Email: mario.caironi@iit.it
b) Department of Physics, Politecnico di Milano, Piazza Leonardo da Vinci, 32, 20133 Milano, Italy.
c) Department of Electronics, Information and Bioengineering, Politecnico di Milano, Piazza Leonardo da Vinci, 32, 20133 Milano, Italy.
d) Smart Materials, Istituto Italiano di Tecnologia, Via Morego 30, 16163, Genova, Italy.
e) Institute for Physical Chemistry, Heidelberg University, 69120, Heidelberg
f) Department of Material Science, Università degli Studi di Milano-Bicocca, via Cozzi, 55, 20125, Milano
g) Department of Engineering for Innovation, University of Salento, Via per Monteroni, 73100 Lecce, Italy

‡ These authors contributed equally

(a)

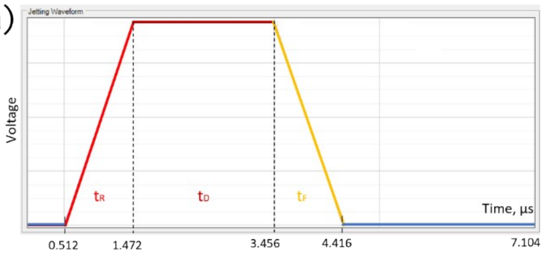

(b)

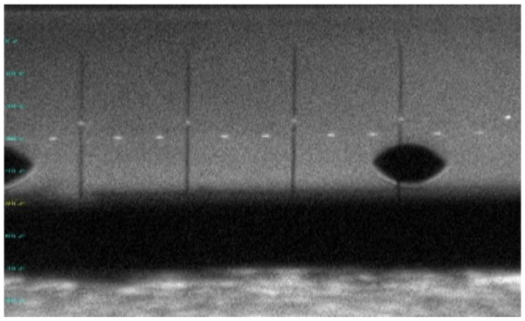

Fig.S1. Throughout this work, a FujiFilm Dimatix DMP-2831 piezoelectric inkjet printer was used. (a) The jetting waveform adopted to print the gold ink was optimized for the 2.4 pl Dimatix Samba cartridge by varying and adapting pulse duration and magnitude of the voltage applied to the piezoelectric actuators. A single pulse voltage waveform was employed. The maximum jetting frequency was set to 5 kHz, and the jetting voltages of ~ 37 V were typically chosen

in order to have a good jetting stability. To ensure the proper wetting of the gold ink and regularity of the printed pattern, the substrate plate of the printer was set to 60 °C. (b) Droplet formation of the gold ink.

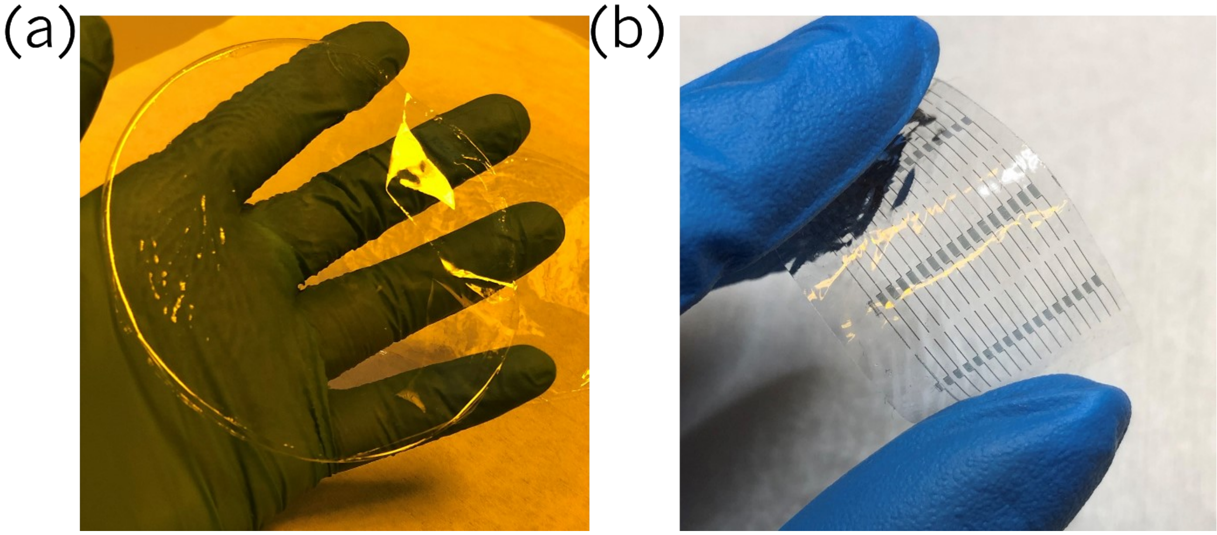

Fig.S2. Photo of (a) ethylcellulose substrate and (b) ethylcellulose substrate with printed gold electrodes on top.

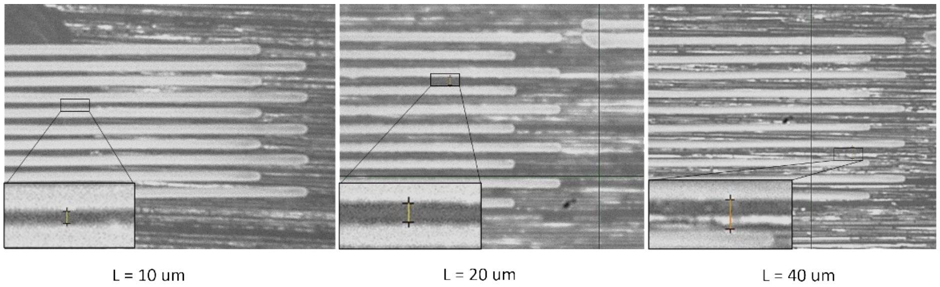

Fig.S3. Gold interdigitated electrodes inkjet-printed on PEN substrate. Control of the channel length *L* down to 10 µm is shown. The picture was taken using the fiducial camera of the Dimatix printer (Drop spacing 20 µm; Jetting voltage 37 V; Substrate temperature 60 °C). The inkjet-printing of the gold ink onto PEN substrates was carried out without any additional surface treatment.

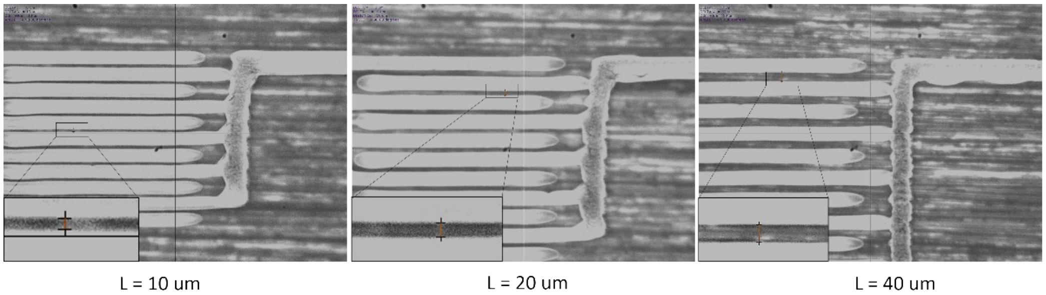

Fig.S4. Gold interdigitated electrodes inkjet-printed on a glass substrate. Control of the channel length *L* down to 10 µm is shown. The picture was taken using the fiducial camera of the Dimatix printer (Drop spacing 15 µm; Jetting voltage 37 V; Substrate temperature 60 °C). The glass substrate required 7 min of oxygen plasma prior the gold ink deposition to ensure the proper continuity of the pattern.

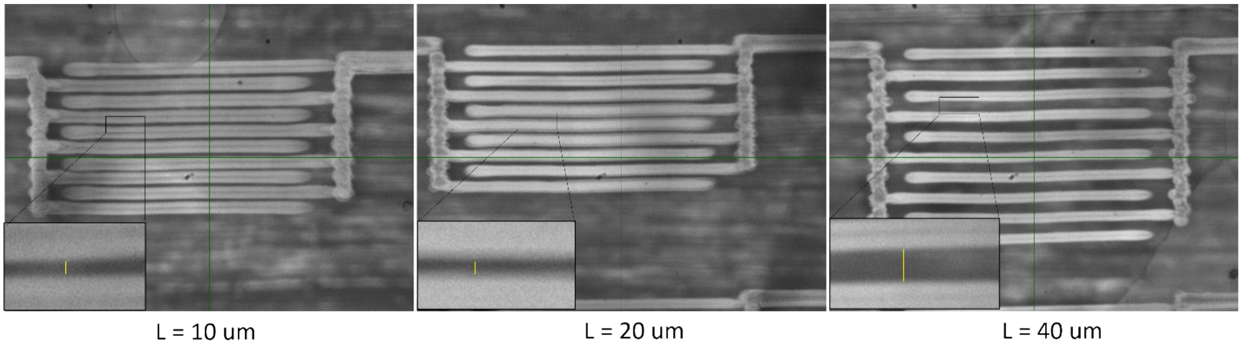

Fig.S5. Gold interdigitated electrodes inkjet-printed on edible ethylcellulose substrate. Control of the channel length *L* down to 10 µm is shown. The picture was taken using the fiducial camera of the Dimatix printer (Drop spacing 20 µm; Jetting voltage 39 V; Substrate temperature 60 °C). The substrate required 1 min of oxygen plasma to ensure a proper ink surface wetting.

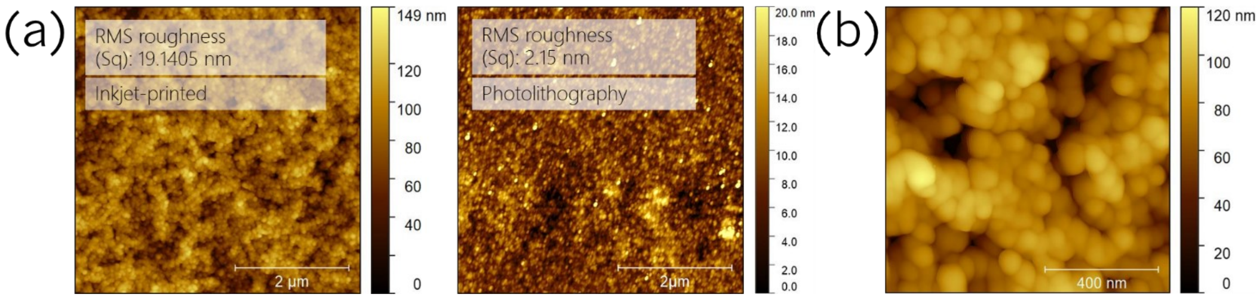

Fig.S6. (a) Comparison of AFM topographic images of the top surfaces of (left) inkjet-printed and (right) photolithographically-patterned gold electrodes on PEN substrate; the image area is 5x5 µm^2. (b) Top surface of inkjet-printed gold electrodes; the image area is 1x1 µm^2.

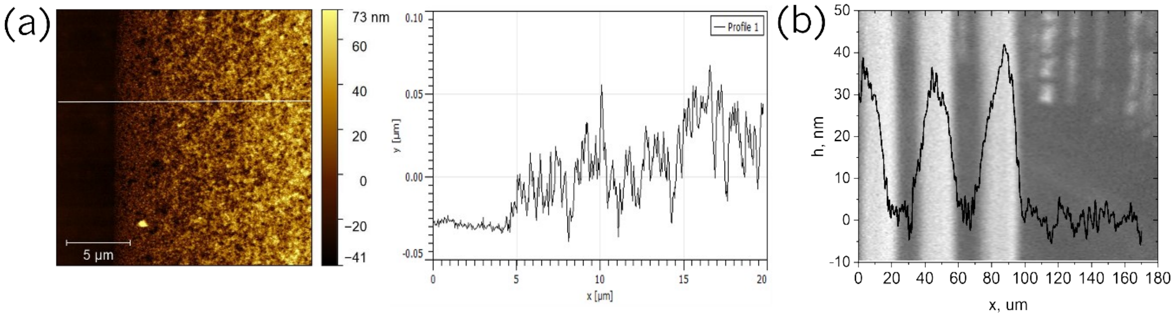

Fig.S7. (a) AFM image and corresponding profile of the boundary region of the gold film inkjet-printed on PEN substrate. The area of the image is 20x20 µm^2. (b) Thickness profile of three gold traces obtained using optical profilometer.

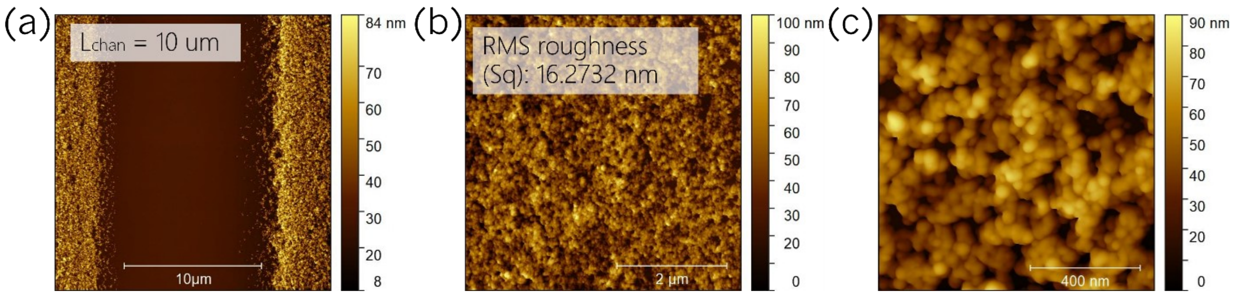

Fig.S8. AFM of gold electrodes inkjet-printed on glass substrate after the plasma treatment. (a) The morphology of the area between the two conducting fingers of the interdigitated electrodes with a channel length L = 10 µm; the area of the image is 20x20 µm^2. (b, c) Topographic images of the top surface of the gold electrode. The image areas are 5x5 µm and 1x1 µm, correspondingly. The edge of the inkjet-printed gold electrodes is not sharp due to the wetting nature of the ink on the glass surface treated with oxygen plasma.

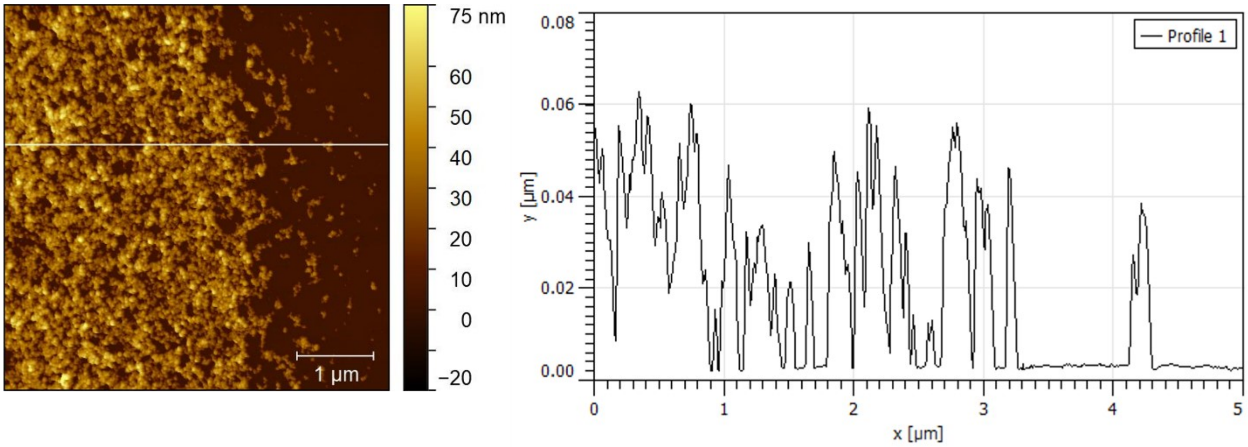

Fig.S9. AFM image and corresponding thickness profile of the boundary region of the gold film inkjet-printed on glass substrate. The area of the image is 5x5 µm.

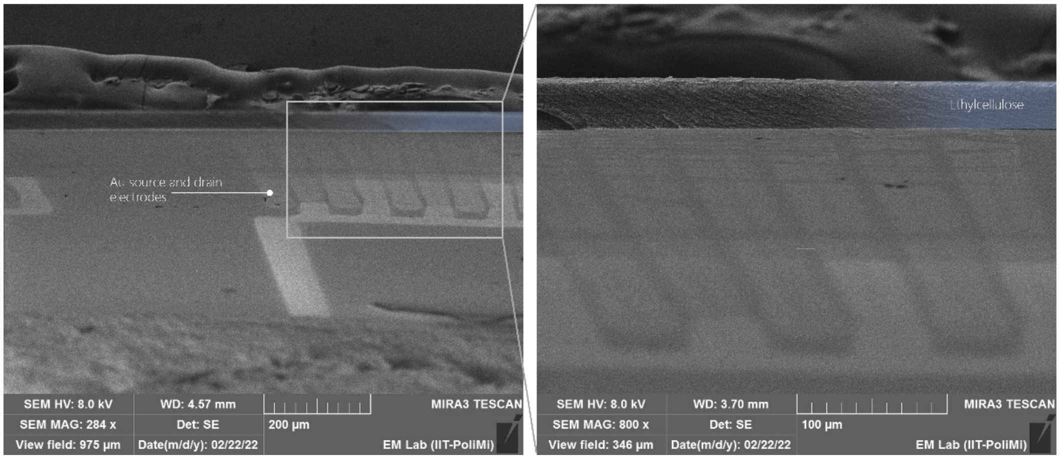

Fig.S10. The tilted view SEM images of gold interdigitated electrodes with L = 10 µm inkjet-printed on edible ethyl cellulose substrate.

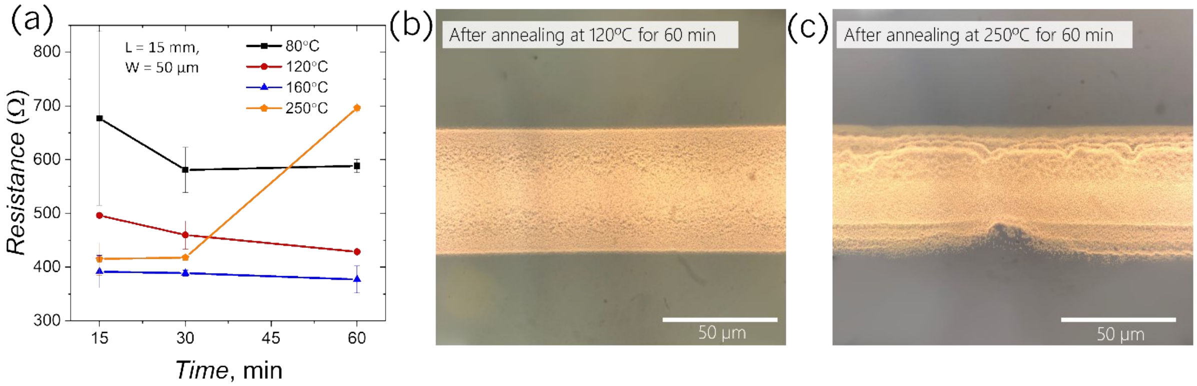

Fig.S11. (a) Dependence of electrical resistance of inkjet-printed gold lines on drying conditions (several combinations of annealing temperature and time). The plots are presented for lines of 15 mm length. Each point corresponds to the average of values measured over 7 samples. The optical microscope image of the gold traces annealed (b) at 120 °C for 60 minutes and (c) at 250 °C for 60 minutes.

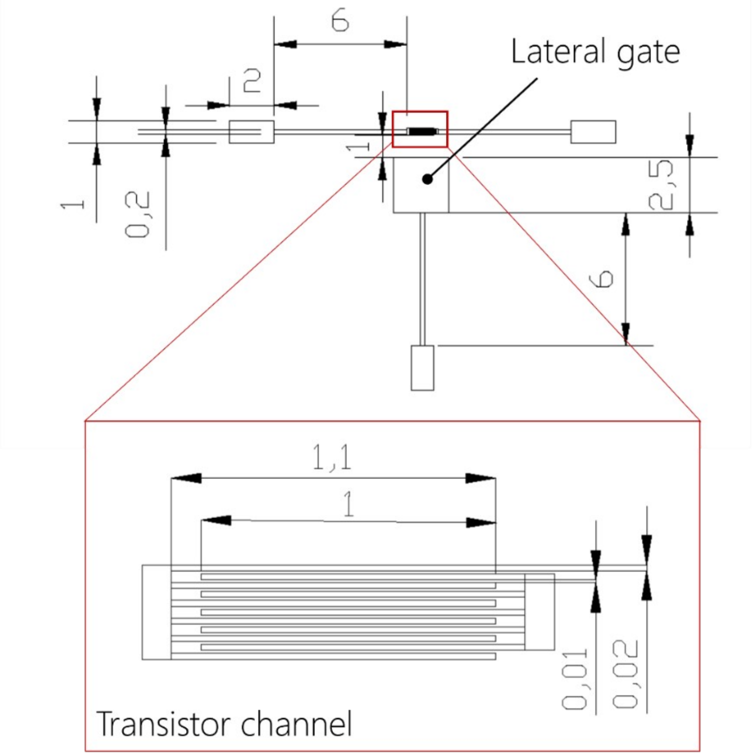

Fig.S12. In-scale AutoCad drawing of the pattern used for the definition of the gold contacts used for the realization of WGTFTs, through both lithography and inkjet-printing. All measurements are in [mm].

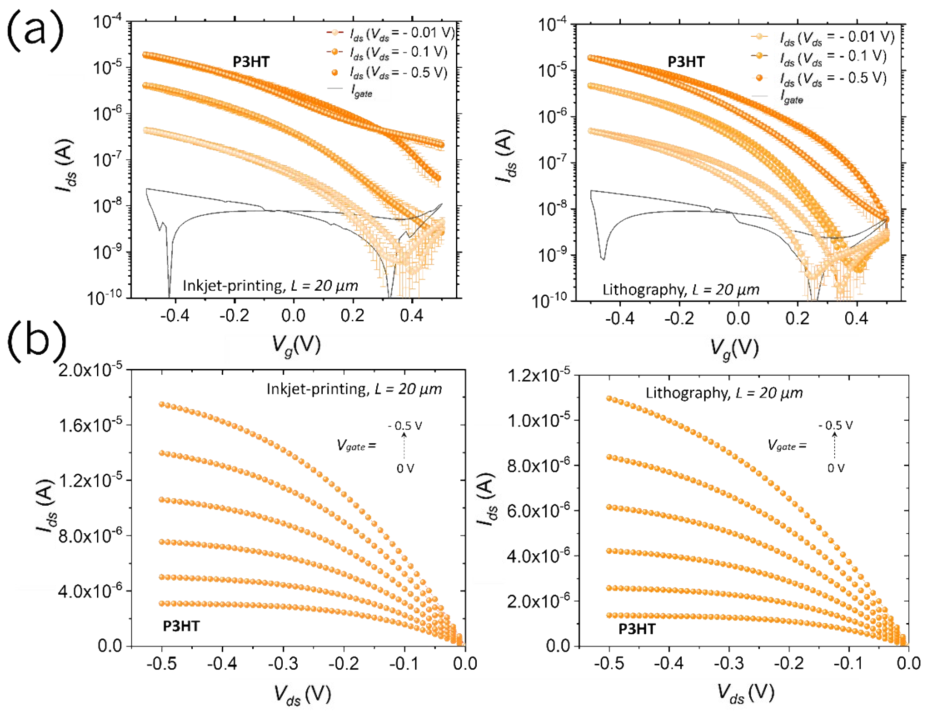

Fig.S13. (a) Transfer characteristic curves for P3HT-based WGOFETs, based on (left) inkjet-printed gold electrodes and (right) photolithographically patterned gold electrodes in linear (Vds = - 0.01 V; - 0.1 V) and saturation (Vds = - 0.5 V) regimes; Vgate sweep rate was 10 mV/s; Transfer curves represent the mean among 7 samples; OFETs geometrical parameters: L =20 µm; W = 10,000 µm. (b) Output characteristic curves for P3HT-based WGOFETs, based on (left) inkjet-printed gold electrodes and (right) photolithographically patterned gold electrodes.

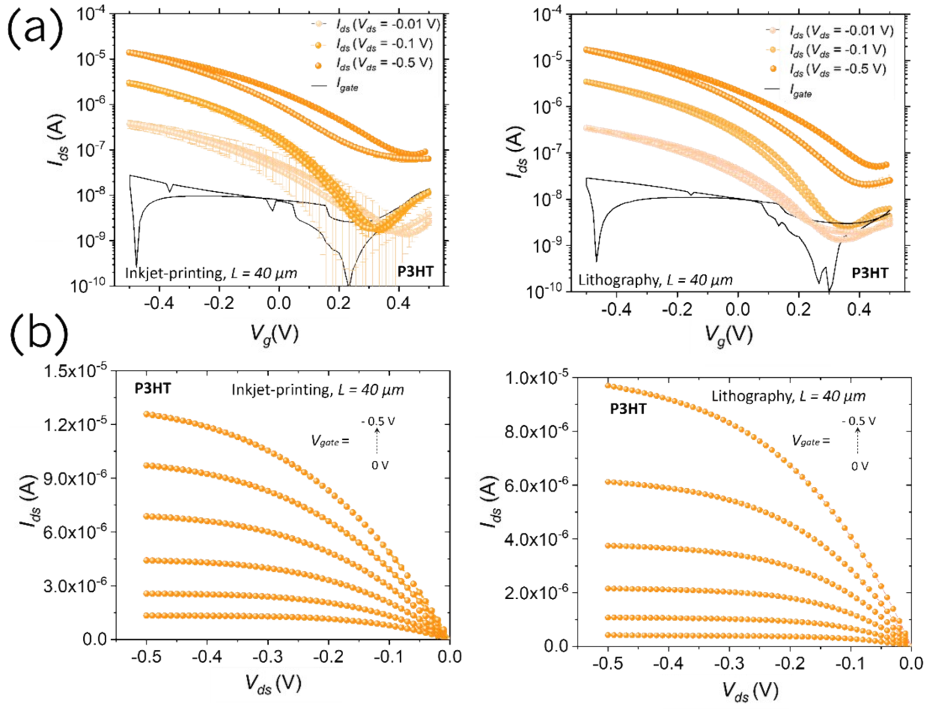

Fig.S14. (a) Transfer characteristic curves for P3HT-based WGOFETs, based on (left) inkjet-printed gold electrodes and (right) photolithographically patterned gold electrodes in linear (Vds = - 0.01 V; - 0.1 V) and saturation (Vds = - 0.5 V) regimes; Vgate

sweep rate was 10 mV/s; Transfer curves represent the mean among 7 samples; OFETs geometrical parameters: L =40 µm; W = 10,000 µm. (b) Output characteristic curves for P3HT-based WGOFETs, based on (left) inkjet-printed gold electrodes and (right)

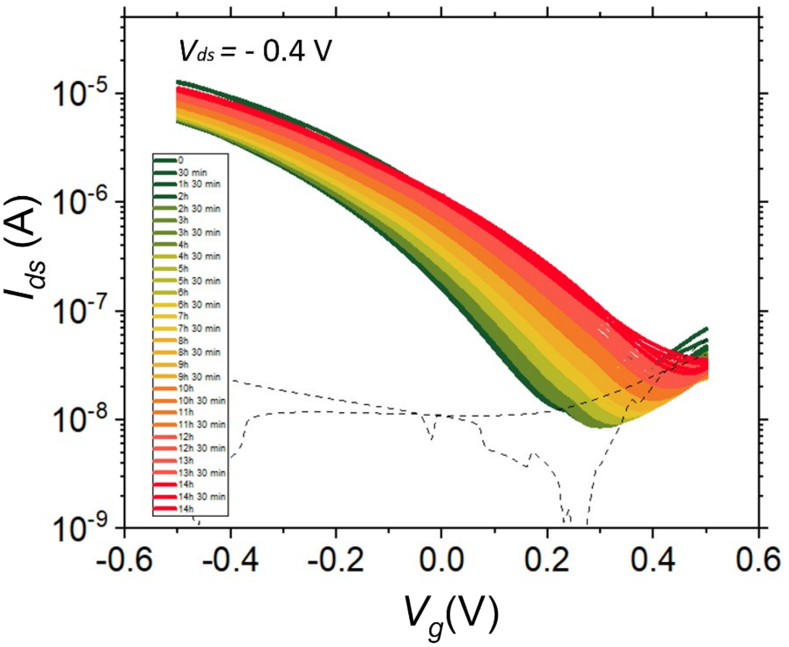

photolithographically patterned gold electrodes.

Fig.S15. The stability of P3HT-based WGTFTs based on inkjet-printed gold electrodes when operating in water. A series of consecutive transfer characteristic curves acquired with 30 min intervals for a duration of 14 h.

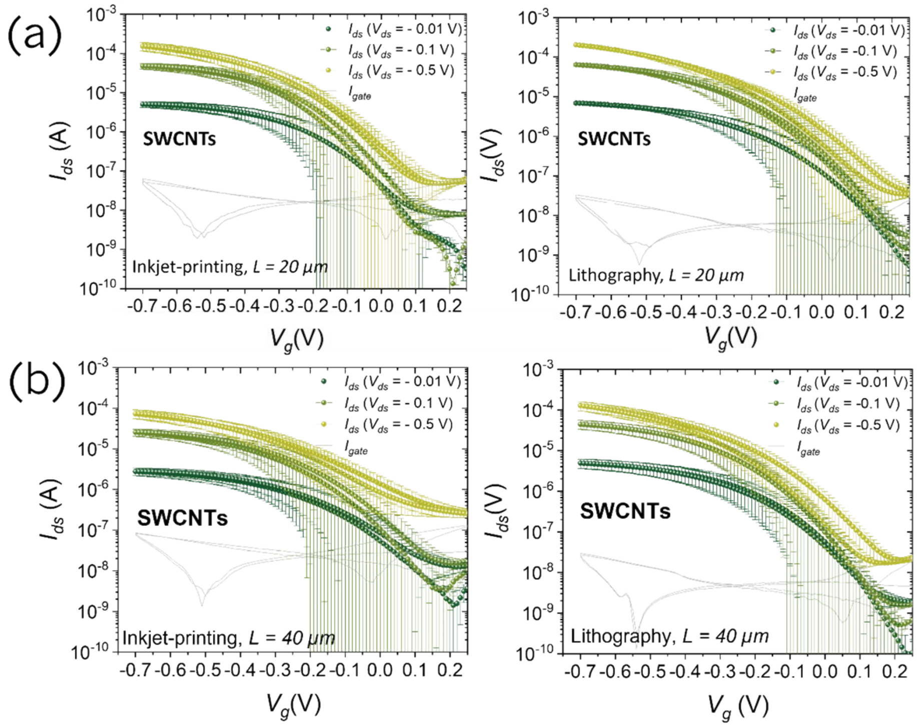

Fig.S16. Transfer characteristic curves for SWCNTs-based WGOFETs, based on (left) inkjet-printed gold electrodes and (right) photolithographically patterned gold electrodes in linear (V_{ds} = - 0.01 V; - 0.1 V) and saturation (V_{ds} = - 0.5 V) regimes; V_{gate} sweep rate was 10 mV/s; Transfer curves represent the mean among 7 samples; (a) OFETs geometrical parameters: L =20 µm; W = 10,000 µm; (b) OFETs geometrical parameters: L =40 µm; W = 10,000 µm.

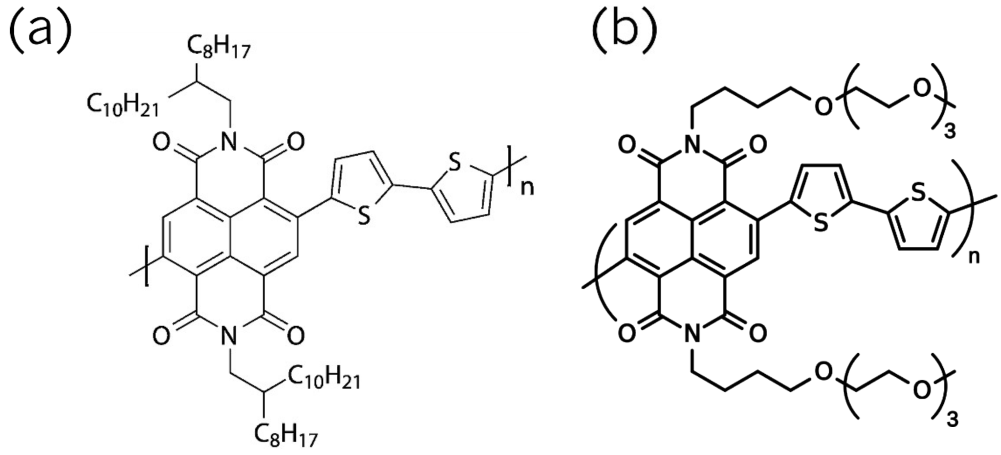

Fig.S17. A molecular structure of semiconductive (a) P(NDI2OD-T2); (b) P(NDI-C4-TEGMe-T2), a modified P(NDI2OD-T2) with a single glycol side chain.

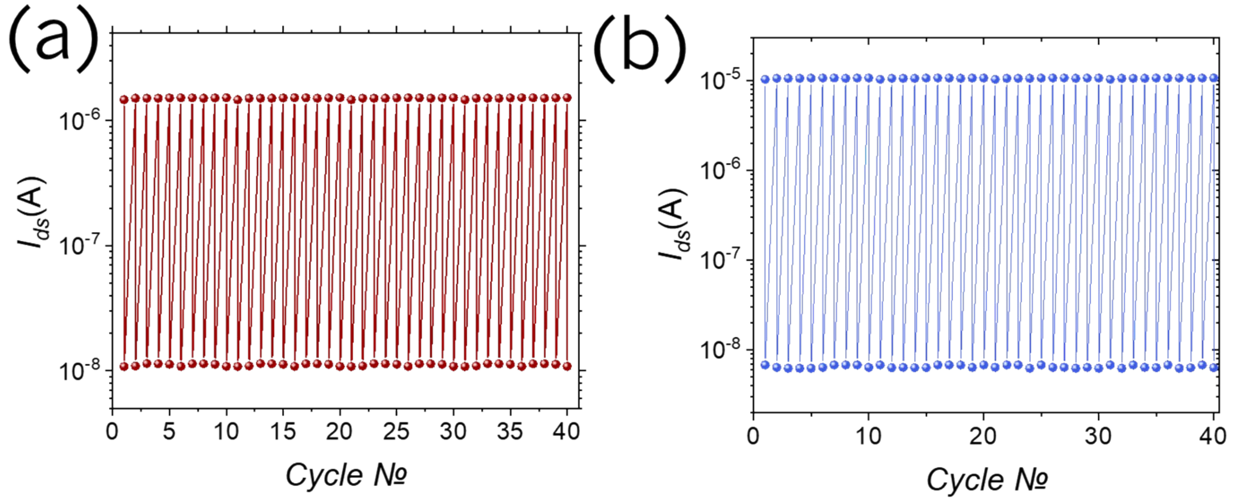

Fig.S18. Operational stability of the fully printed Chitosan-Gated Transistors under continuous cycling voltage test in air (cycle duration 40 s, total test duration 30 min). The test was carried out by switching (a) the p-type device on (Vgate = -1 V, Vds = - 0.2 V;) and off (Vgate = 0 V, Vds = 0.2 V) and (b) n-type on (Vgate = 1 V, Vds = 0.2 V;) and off (Vgate = 0 V, Vds = - 0.2 V). No evidence of devices degradation was observed.

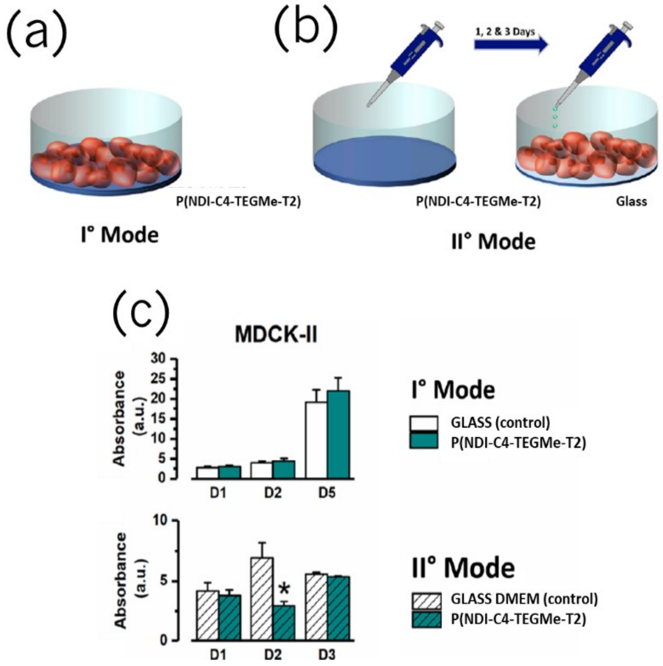

Fig.S19: Cytotoxicity tests of p(NDI-C4-TEGMe-T2). MDCK-II were cultured in cell culture flasks containing Dulbecco's modified Eagle's medium (DMEM) with 10% fetal bovine serum (Sigma Aldrich), 2mM glutamine (Sigma Aldrich), 100 U/ml streptomycin (Sigma Aldrich) and 100 U/mL penicillin (Sigma Aldrich). Cells were maintained in humidified atmosphere at 37 °C with 5% CO_2. At 80-90% of confluence, cells were enzymatically detached using 0.5% trypsin-0.2% EDTA (Sigma Aldrich) for 5min and plated for the cell viability assay. 2mg/ml of fibronectin was used as coating in all experiments. The Alamar Blue (Invitrogen) assay was used to evaluate the cytotoxicity of p(NDI-C4-TEGMe-T2).Cells were incubated with culture medium and 1% of Almar Blue for 3 hours at 37°C, then 100 µl was collected for each condition and pipetted into a 96-well plate. Florescence was read on TECAN Spark10M automated plate-reading fluorometer, using an excitation of 540 nm and an emission of 600 nm. The florescent emission at 600 nm is an index of the viability and capability to proliferate. All samples were processed in triplicate and normalized for a background value. The p(NDI-C4-TEGMe-T2) toxicity was tested in two modes. In the first one, the MDCK-II cells were grown on the polymer film, whereas in the second mode the culture medium was exposed to p(NDI-C4-TEGMe-T2) for few days and subsequently administered to the cells. The cell viability was tested after 1,2,3 or 5 days after the treatments in both conditions. Concerning MCDK-II proliferation, there were no differences between p(NDI-C4-TEGMe-T2) samples and control in the first condition while, in the second one, at day 2 cells plated with PNDI2-DMEM showed a reduction as compared with control. This effect was not present at day 3, suggesting that PNDI2 caused a delay in the proliferation rate but didn't stop it, indicating therefore no sign of cytotoxicity.

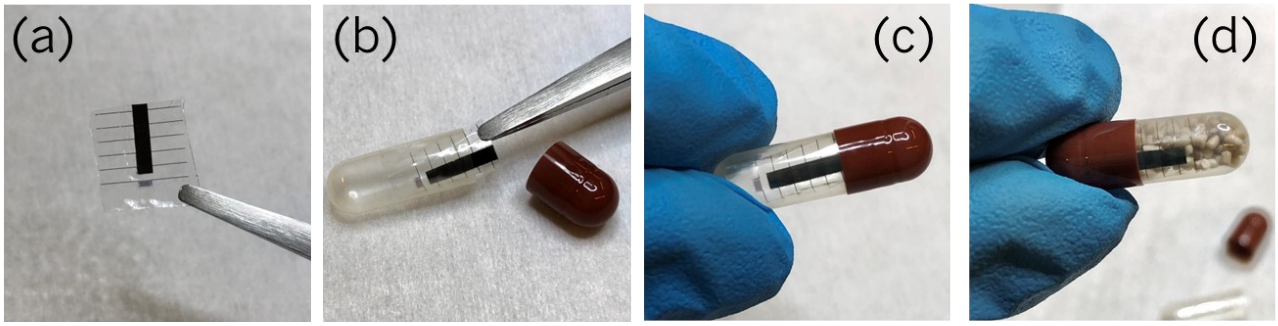

Fig.S20: Flexible, fully printed Chitosan-gated transistors. (a) Digital photo of the devices (6 transistors) on an edible, stretchable ethyl cellulose substrate, allowing the devices to being folded to fit inside a gelatin capsule commonly used for drug delivery (b), (c). (d) The final capsule containing both the edible transistors and the drug molecules.

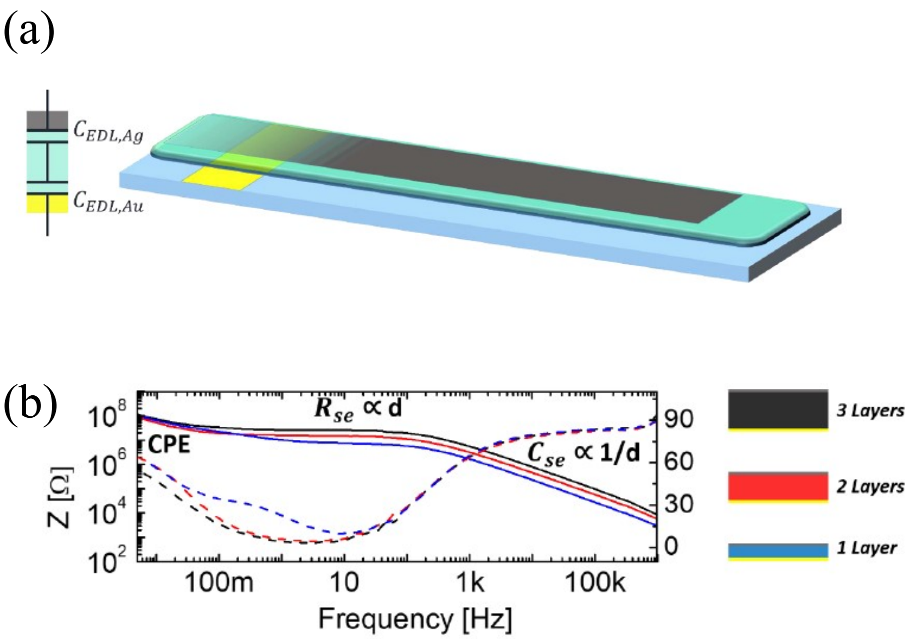

Fig.S21: electrochemical impedance spectroscopy (EIS) was performed[1] on samples realized by drop-casting an increasing number of chitosan layers on top of each other, sandwiched between two metallic layers (a). The results are reported in the Bode plot (b), where the impedance spectra are analyzed for 3 samples in the 10mHz – 1MHz frequency range. A capacitive component scaling as 1/d can be identified at high frequencies, while the ionic resistance scales proportionally with the thickness of the electrolyte. A constant phase elements (CPE) component is present in the low frequency range, and can be ascribed to the formation of double layers at the interfaces between the chitosan blend and the two metallic electrodes. Being a surface phenomenon, it does not scale with the thickness of the film, as expected. From this region, an extracted capacitance of ≈ 8 μF/cm^2 was calculated

1. Melloni, F. Implantable and Ingestible Devices : New Opportunities for Electronics and Human Body Interaction. (2020).
